# Skin of Color Representation Trends in JAAD Case Reports 2015-2021: Content Analysis

**DOI:** 10.2196/40816

**Published:** 2023-06-02

**Authors:** Nathaniel A Marroquin, Alexa Carboni, Morgan Zueger, Mindy D Szeto, Jessica Kirk, Jieying Wu, Hamza Ajmal, Robert P Dellavalle

**Affiliations:** 1 Rocky Vista University College of Osteopathic Medicine Parker, CO United States; 2 Department of Dermatology University of Colorado Anschutz Medical Campus Aurora, CO United States; 3 Dermatology Service US Department of Veterans Affairs Rocky Mountain Regional Medical Center Aurora, CO United States

**Keywords:** skin of color, case report, diversity, diverse, equity, inclusion, representation, skin tone, image classification, case photo, case image, racism, skin color, race, racial, skin

Underrepresentation of skin of color (SoC) in academic resources may curtail diagnostic training and exacerbate health disparities given an increasingly diverse US population [1]. Case reports are important starting points and foundational for high-quality studies in the research pyramid evidence hierarchy [2], thus inclusivity and diverse representation should be encouraged. We therefore sought to examine SoC representation and race/ethnicity reporting in all case photos published by *JAAD Case Reports* since its inception in 2015 through 2021.

Skin tones represented by each available case photo were assessed by two independent blinded reviewers with dermatology experience and recorded as either light (corresponding to Fitzpatrick I-II), medium (III-IV), or dark (V-VI) [3], with a third independent reviewer resolving any discrepancies prior to analysis. Case author–reported race/ethnicity was tabulated as White, Black, Hispanic, Asian, or other and was compared to the case image if one was presented.

A total of 2451 cases were reviewed. In 2015, images were perceived as 73% light, 15% medium, and 12% dark skin toned (Figure 1). Percentages of light skin tones decreased to 59% from 2015 to 2021 (chi-square *P*=.008), corresponding with increasing percentages of dark- and medium-toned images. Total cases that reported any race/ethnicity decreased from 40% in 2015 to 24% in 2021 (*P*<.001), and of those fractions, the proportion of White race reported largely remained equal (~50%) to that of Black, Hispanic, Asian, and other combined. Patients with light skin tones were more commonly reported by case authors as White, and patients with dark skin tones were more frequently reported as Black. However, ~65% of cases that did not include a corresponding image were reported as White (Figure 2).

While *JAAD Case Reports* continues to publish light skin tones more frequently, trends toward increasing SoC representation are promising. Interestingly, the frequent omission of photos among White case report participants could suggest that authors and editors perceive image necessity differently for White patients compared to patients of other races. Without further explanation of image omission, this presents a challenge for the field in preventing unconscious bias and the erroneous concept of race as a biological construct [4]. Indeed, a previous 2018-2020 analysis of 52 dermatology journals revealed that only 16.3% of publications on average were focused on diversity or SoC, perhaps related to ongoing disparities in access to dermatologic care among SoC populations [5]. Given that case reports are foundational for further research and the urgent need to address underrepresentation and health disparities, we hope to motivate further discussion and urge journals to consider establishing consistent reporting criteria when publishing case reports, whether that requires including or omitting images or race/ethnicity descriptors, for example. In parallel, greater attention should be afforded to the nuances of how bias could potentially be introduced via reporting decisions. Nevertheless, including more examples of conditions appearing on different skin tones can bolster the relevance of case reports in improving clinical care for diverse populations.

**Figure 1:**
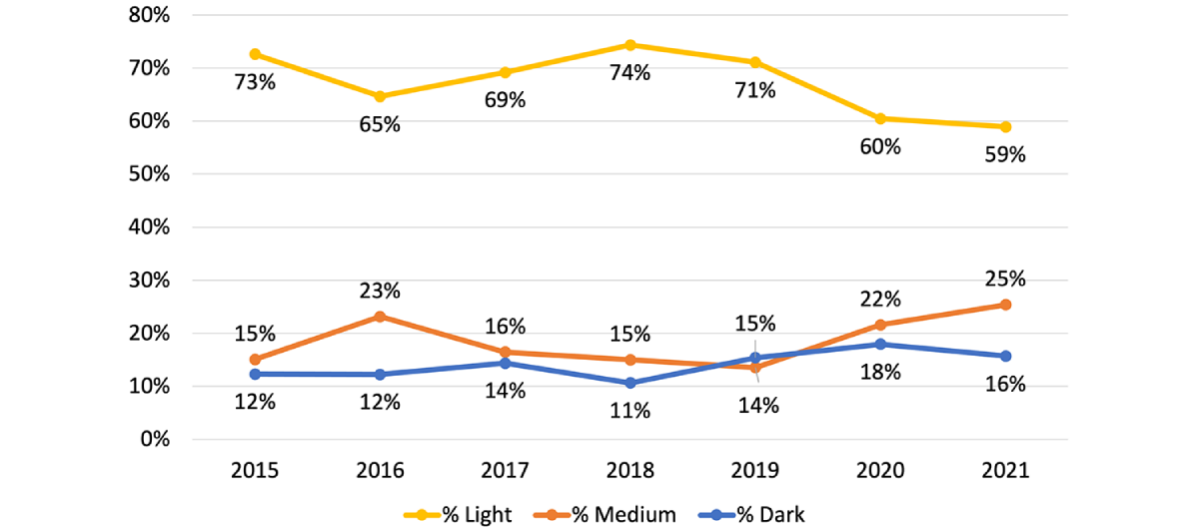
Percentages of perceived skin tones in JAAD Case Report images by publication year, 2015-2021.

**Figure 2:**
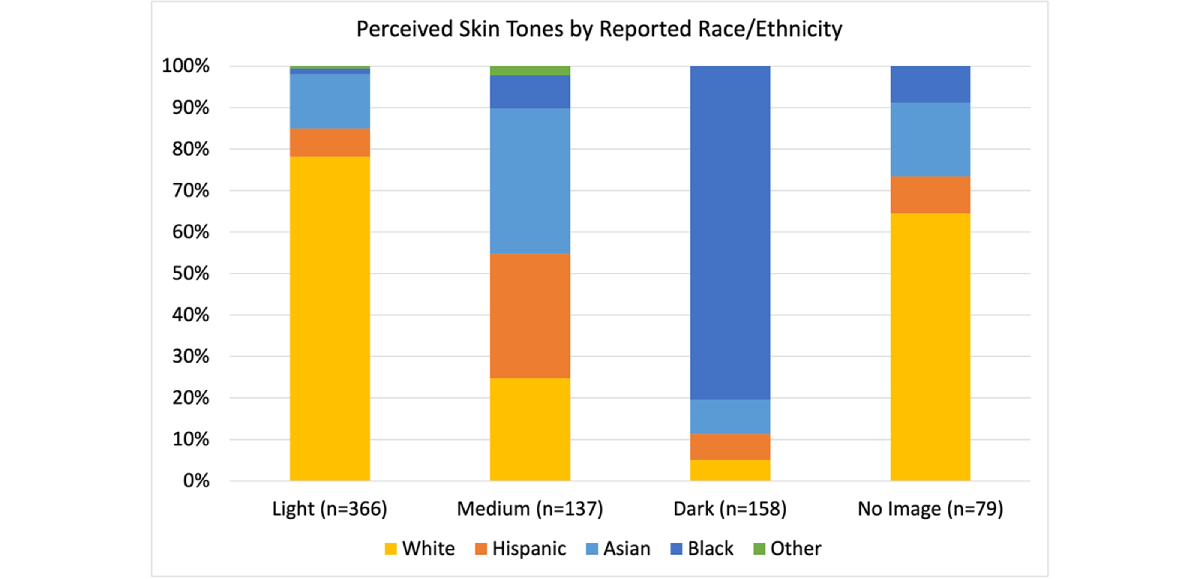
Perceived skin tones for JAAD Case Report images from 2015 to 2021 with reported race/ethnicity.

## Abbreviations

SoC: skin of color

## References

[1] Alvarado SM, Feng H (2021). Representation of dark skin images of common dermatologic conditions in educational resources: a cross-sectional analysis. J Am Acad Dermatol.

[2] Albrecht J, Werth VP, Bigby M (2009). The role of case reports in evidence-based practice, with suggestions for improving their reporting. J Am Acad Dermatol.

[3] Ebede T, Papier A (2006). Disparities in dermatology educational resources. J Am Acad Dermatol.

[4] Huang JT, Davies OMT, Siegel DH (2021). Achieving equity and inclusion in pediatric dermatology research: priorities and considerations. Pediatr Dermatol.

[5] Wilson BN, Sun M, Ashbaugh AG, Ohri S, Yeh C, Murrell DF, Murase JE (2021). Assessment of skin of color and diversity and inclusion content of dermatologic published literature: an analysis and call to action. Int J Womens Dermatol.

